# Jersey number detection using synthetic data in a low-data regime

**DOI:** 10.3389/frai.2022.988113

**Published:** 2022-10-06

**Authors:** Divya Bhargavi, Sia Gholami, Erika Pelaez Coyotl

**Affiliations:** Amazon Web Services, San Francisco, CA, United States

**Keywords:** synthetic data, jersey number detection, low-data regime, convolutional neural network, ensemble model

## Abstract

Player identification is an essential and complex task in sports video analysis. Different strategies have been devised over the years and identification based on jersey numbers is one of the most common approaches given its versatility and relative simplicity. However, automatic detection of jersey numbers is challenging due to changing camera angles, low video resolution, small object size in wide-range shots, and transient changes in the player's posture and movement. In this paper, we present a novel approach for jersey number identification in a small, highly imbalanced dataset from the Seattle Seahawks practice videos. We generate novel synthetic datasets of different complexities to mitigate the data imbalance and scarcity in the samples. To show the effectiveness of our synthetic data generation, we use a multi-step strategy that enforces attention to a particular region of interest (player's torso), to identify jersey numbers. The solution first identifies and crops players in a frame using a person detection model, then utilizes a human pose estimation model to localize jersey numbers in the detected players, obviating the need for annotating bounding boxes for number detection. We experimented with two sets of Convolutional Neural Networks (CNNs) with different learning objectives: multi-class for two-digit number identification and multi-label for digit-wise detection to compare performance. Our experiments indicate that our novel synthetic data generation method improves the accuracy of various CNN models by 9% overall, and 18% on low frequency numbers.

## 1. Introduction

**Figure 1:**
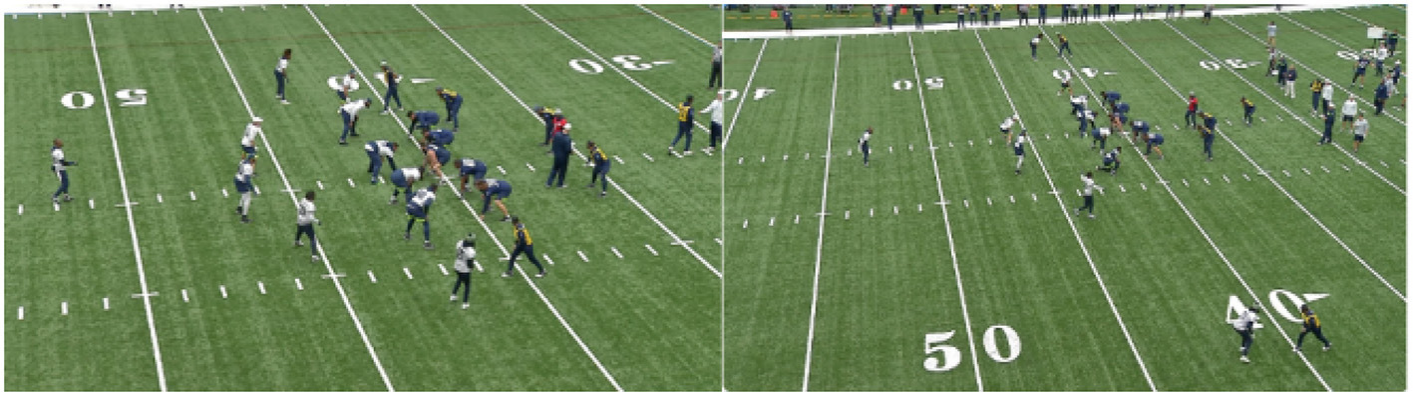
Sample frames from practice videos. The frames are sampled a couple of seconds apart with different camera zoom levels offering varying visibility of the jersey numbers.

**Figure 2:**
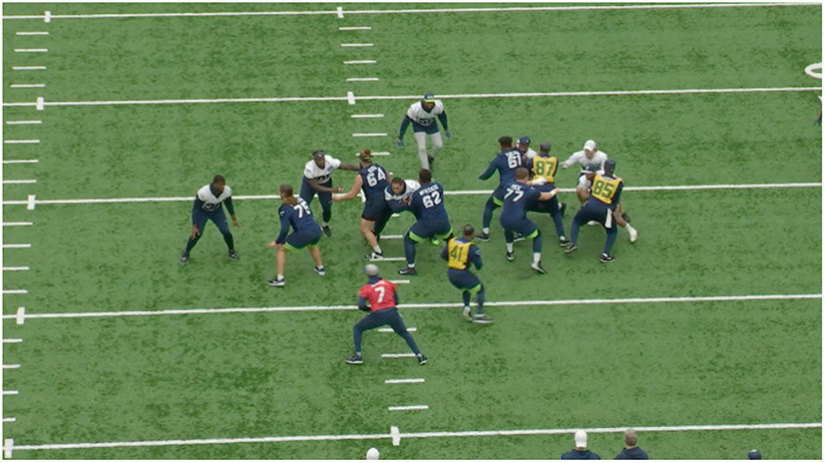
Sample practice video frame with various player poses and orientations.

The interest in analyzing team sport videos has increased significantly in academia and Industry in recent years (Ye et al., 2005; Šari et al., 2008; Lu et al., 2013; Gerke et al., 2015; Li et al., 2018; Liu and Bhanu, 2019; Kröckel and Bodendorf, 2020; Wilson, 2020; Vats et al., 2021). This is essential for sports broadcasters and teams to understand key events in the game and extract crucial information from the videos. Applications and use cases include identifying participating players, tracking player movement for game statistics, measuring health and safety indicators, and automatically placing graphic overlays. For broadcasters and teams that don't have the leeway or the capital to install hardware sensors in player wearables, a Computer Vision (CV) based solution is the only viable option to automatically understand and generate insights from games or practice videos. One important task in all sports CV applications is identifying players, specifically identifying players with their jersey numbers. This task is challenging due to distortion and deformation of player jerseys based on the player posture, movement, and camera angle, rarity of labeled datasets, low-quality videos, small image size in zoomed out videos, and warped display caused by the player movement (see Figures 1, 2).

Current approaches for jersey number identification consist of two steps: collecting and annotating large datasets (Li et al., 2018; Vats et al., 2021), and training large and complex models (Li et al., 2018; Liu and Bhanu, 2019; Vats et al., 2021). These approaches include either sequential training of multiple CV models or training one large model, solving for two objectives: identifying the jersey number location (through custom object detection models or training a custom human pose estimation model) and classifying the jersey number (Gerke et al., 2015; Li et al., 2018; Liu and Bhanu, 2019; Vats et al., 2021). These approaches are tedious, time-consuming, and cost-prohibitive thus making it intractable for all sports organizations.

In this paper, we present a method to detect jersey numbers with a set of relatively small and efficient models. We present a novel approach to synthetic data generation for the task of detecting jersey numbers in a small dataset. Our test dataset consists of practice video footage from the Seattle Seahawks team. To show the effectiveness of our synthetic data generation approach we use a three-step method to number detection leveraging pre-trained models. We first identify and crop players in a video frame using a person detection model. We then utilize a human pose estimation model for localizing jerseys on the detected players using the torso key-points, obviating the need for annotating bounding boxes for number locations. This results in images that are less than 20 × 25 pixel with a high imbalance in jersey numbers (see Figure 4). Finally, we experiment with two sets of learning approaches for model training—multi-class and multi-label each yielding an accuracy of 88%, with an ensemble accuracy of 89% to identify jersey numbers from cropped player torsos.

To compensate for the low number of examples in some of the jersey numbers, we propose two novel synthetic dataset generators—Simple2D and Complex2D. The Simple2D generator creates two-digit number images from different combinations of fonts and background colors to mimic those of the Seattle Seahawks jerseys. The Complex2D generator superimposes the Simple2D numbers on random COCO dataset (Lin et al., 2014) images to add more complexity to the background and make the model training robust. By pre-training our two Convolutional Neural Networks (CNNs) on these synthetic datasets, we observe a 9% increase in accuracy on the ensemble models pre-trained with synthetic data compared to the baseline models trained only on the Seattle Seahawks dataset.

## 2. Related work

### 2.1. Synthetic data generation

Convolutional Neural Network algorithms, that are commonly used in most CV tasks, require large datasets to learn patterns in images. Collecting and annotating large datasets is a manual, costly, and time-consuming task. Several new approaches including Active Learning (Settles, 2009), Zero or Few-shot learning (Larochelle et al., 2008), and Synthetic data generation (De Campos et al., 2009) have emerged in recent years to tackle complexities in obtaining a large annotated dataset. Our work focuses primarily on the use of synthetically generated data. This idea dates back to the 1990's (Nikolenko, 2021b) and is an active field of research that alleviates the cost and efforts needed to obtain and manually label real-world data. Nowadays, models (pre)-trained on synthetic datasets have a broad range of utility including feature matching (DeTone et al., 2018) autonomous driving (Siam et al., 2021), robotics indoor and aerial navigation (Nikolenko, 2021a), scene segmentation (Roberts et al., 2021), and anonymized image generation in healthcare (Piacentino et al., 2021). The approaches broadly adopt the following process: pre-train with synthetic data before training on real-world scenes (DeTone et al., 2018; Hinterstoisser et al., 2019), generate composites of synthetic data and real images to create a new one that contains the desired representation (Hinterstoisser et al., 2018) or generate realistic datasets using simulation engines like Unity (Borkman et al., 2021) or generative models like GANs (Jeon et al., 2021; Mustikovela et al., 2021). There are limitations to each of these regimes but one of the most common pitfalls is performance deterioration in real-world datasets. Models trained only synthetic datasets don't generalize to real-world data; this phenomenon is called “domain shift” (Jeon et al., 2021).

In order to reduce the need for annotating large dataset as well as account for the size and imbalance of the real-world data, we generated two double-digit synthetic datasets—Simple2D and Complex2D with different levels of complexity as described in section 3.2.2. This helps to circumvent the domain shift when only synthetic data is used and improves generalization on real-world data for fine-tuning.

### 2.2. Number identification

Automatic number identification in sports video has evolved from classical CV techniques including feature extraction using contrast adjustment, edge detection of numbers (Ye et al., 2005; Šari et al., 2008; Lu et al., 2013) to deep learning-based architectures that use CNNs for classification (Gerke et al., 2015; Li et al., 2018; Liu and Bhanu, 2019; Vats et al., 2021). A fundamental problem in number identification in sports is the jersey number distortion due to erratic and continuous player movement. The spatial transformer-based approach introduced in Li et al. (2018) tries to localize and better position the number, so that the classifier has a better chance of an accurate prediction. The faster-RCNN with pose estimation guidance mechanism (Liu and Bhanu, 2019) combines the detection, classification, and key-point estimation tasks in one large network to correct region proposals, reducing the number of false negative predictions. This approach needed careful labeling of the player bounding-boxes and four human body key-points, shoulder (right, left), hip (right, left), in addition to the numbers. It also made use of high-resolution number images (512 pixel). This approach yields 92% accuracy for jersey number recognition as a whole and 94% on the digit-wise number recognition task. However, getting the right conditions for it i.e., label the dataset for the three tasks, acquiring high resolution images, and training a large model might be challenging for real-world cases. Furthermore, a lack of standardization and availability of public (commercial use) datasets, makes it difficult to obtain a benchmark for the jersey number identification task.

## 3. Approach

### 3.1. Task definition

We define a jersey number as the one or two-digit number printed on the back of a player's shirt. The jersey number is used to identify and distinguish players and one number is associated with exactly one player. Our solution takes cropped images of player's torsos as input and attempts to classify the jersey number into 101 classes (0–99 for actual numbers and 100 for unrecognizable images or jerseys with no numbers).

### 3.2. American football dataset

**Figure 3:**
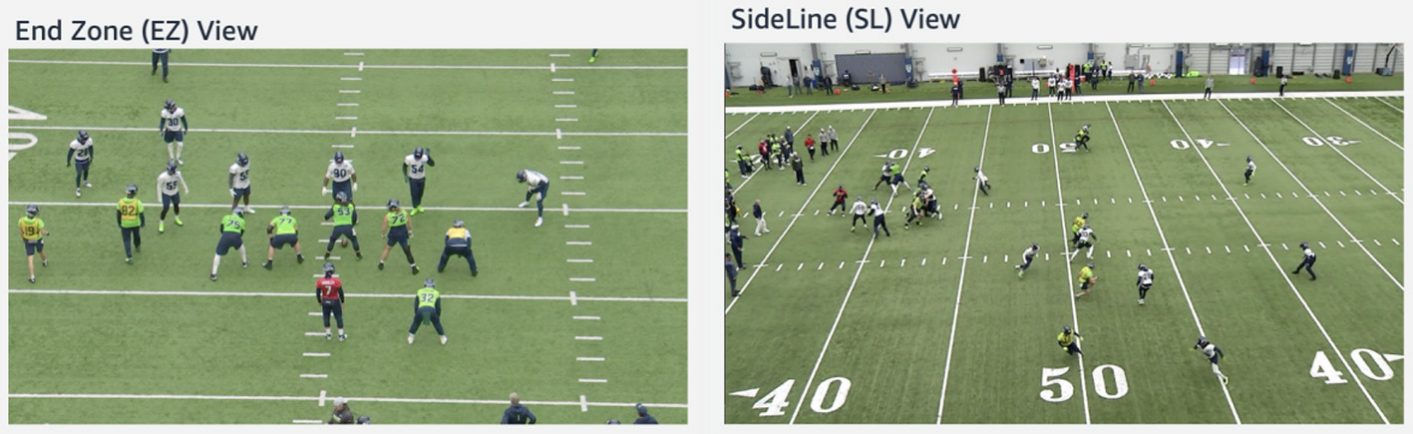
Examples of frames obtained from the two different angles from the training videos. Left, is the endzone view of the players. Right, is the sideline view which offers better visibility into jersey numbers. Within a play, we can find players, observers with/without football jerseys.

The dataset used for this work consisted of a collection of six practice videos from two angles for training and additional four practice videos for testing from the Seattle Seahawks archives. Half of the videos were from the endzone perspective, the scoring zone between the end line and the goal line, the other half were from the sideline perspective, the boundary line that separates the play area from the sides. Both cameras were placed on a high altitude to get a panoramic view for the play and capture the majority of the actions taken by the players. A pitfall for collecting data using this camera angle is that the size of a player is less than 10% of the image size when the players are far away from the camera. In addition, the sideline view has restricted visibility of jersey numbers compared to end-zone (see Figure 3). The videos were recorded in 1,280 × 720 resolution and we sampled frames from each video at 1, 5, and 10 frames per second (fps) rates. We noticed that images sampled at 5 fps sufficiently captured all the jersey numbers in a play and we decided to use the same sampling rate throughout our solution.

#### 3.2.1. Jersey number localization

To mitigate the need for annotating player location, jersey number bounding boxes and consequently training person and jersey number detection models, we used pre-trained models for person detection and pose estimation to localize the jersey number region. This approach prevents the model from generating correlations with wrong features including player background, helmets or clothing items, and confining the learning to the region of interest.

**Figure 4:**
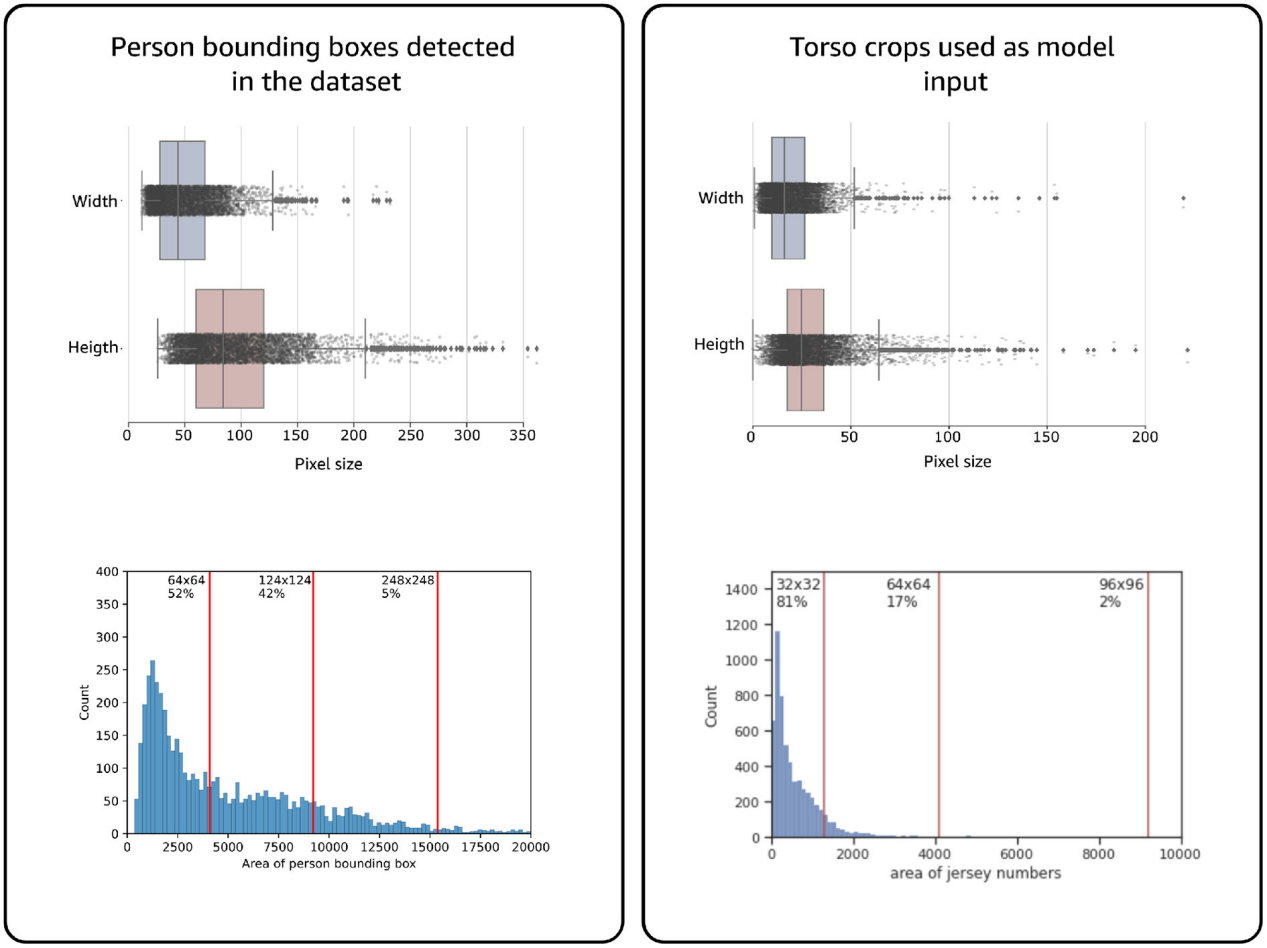
Distribution of the sizes from person and torso bounding boxes. Note how the great majority of torso sizes is less than 32 × 32 pixel.

For the number localization we first used a pre-trained person detector, Centernet (Duan et al., 2019) model (ResNet50 backbone), to detect and crop players from an image. Instead of training a custom human key-point estimation head (Liu and Bhanu, 2019), we use a pre-trained, pose estimation model, AlphaPose, to identify four torso key-points (left and right—hips and shoulders) on the cropped player images from the person detection step (see Figure 7). We use the four key-points to create a bounding box around jersey numbers. To accommodate inaccuracies in key-point prediction and localization due to complex human poses, we increased the size of torso keypoint area by expanding the coordinates 60% outward to better capture jersey numbers. The torso area is then cropped and used as the input for the number prediction models discussed in section 3.2.2. In previous works, the use of high-resolution images of players and jersey numbers are very common. However, the videos in our dataset were captured from a bird's eye view, where jersey numbers were smaller than 32 × 32 pixel. In fact, the average size of the torso crops is 20 × 25 with the actual jersey number being even a smaller portion of this area (see Figure 4).

**Figure 5:**
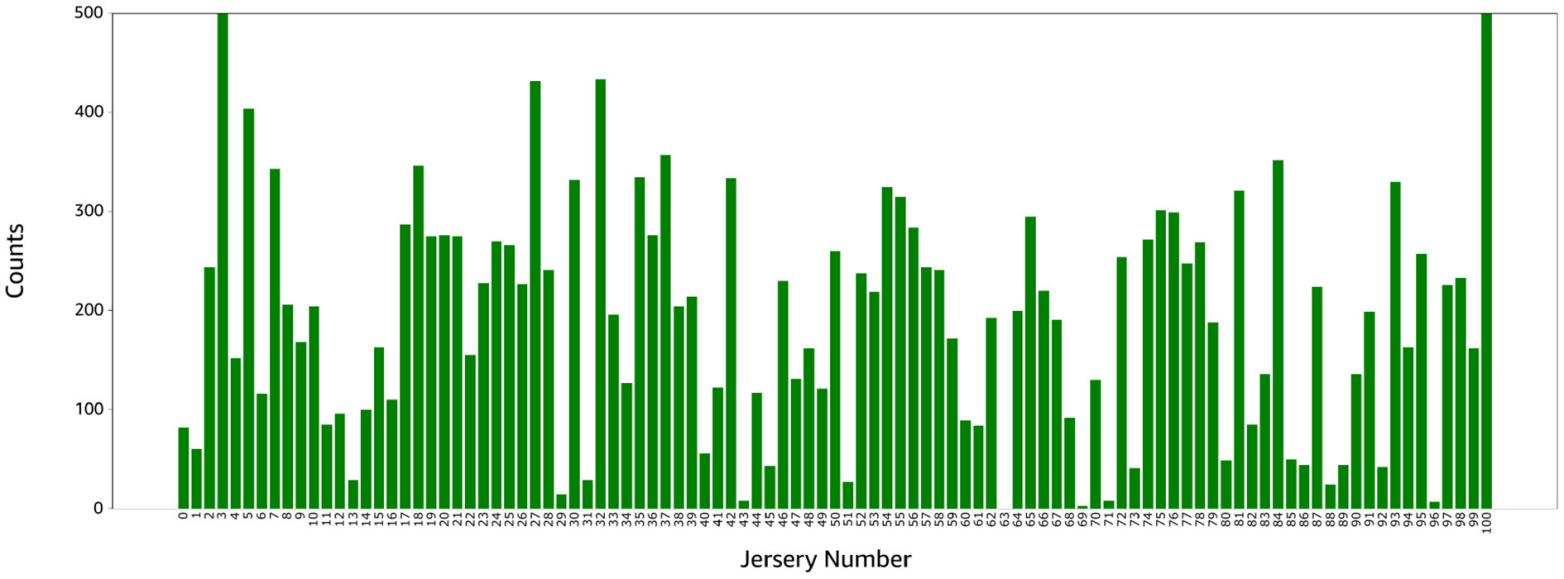
Distribution of the jersey number labels in the training set. Number 3 has 500+ images while numbers 43, 63, 69, and 93 have 10 images or less.

After player detection and jersey number localization, we generated 9,000 candidate images for number detection. We labeled the images with Amazon SageMaker GroundTruth and observed that 6,000 images contained non-players (trainers, referees, watchers); the pose estimation model for jersey number localization simply identifies human body key-points and doesn't differentiate between players and non-players. 3,000 labeled images with severe imbalance (see Figure 5) were usable for the training.

#### 3.2.2. Synthetic data generation

In previous works, a licensed (SVHN— Goodfellow et al., 2013) or a large custom dataset is used for (pre)-training number recognition models. We initially investigated the use of two-digit MNIST (Sun, 2019), however it did not have pixel color and font variations needed for jersey detection and performed poorly in our tests. Since there are no standardized public datasets with permissive licenses, we created two two-digit synthetic datasets to pre-train our models; a simple two-digit (Simple2D) numbers with font and background similar to the football dataset and other with two-digit synthetic numbers superimposed on COCO (Lin et al., 2014) dataset images (Complex2D) to account for variations in numbers background.

The Simple2D dataset was generated by randomly selecting a number from a uniform distribution of 0–9 and scaling it by a random factor. Color backgrounds (Red, Navy Blue, Green, Red, Yellow, White) and special font (Freshman ) that resembled the team jerseys were used to generate these numbers (see Figure 4). One Light, five Medium, and five Hard augmentations (see Table 1) were used on each digit to be later permuted and concatenated to obtain 4,000 images (100 × 100 pixel) of each two-digit number, from 00 to 99. The resulting dataset consisted of a total of 400,000 images.

**Table 1:** Synthetic simple 2D data augmentation levels.

**Name**	**Augmentations**
Light	Gaussian noise, optical distortion
Medium	Light + grid distortion
Hard	Medium + shuffling RGB channels, random shift-scale-rotation

**Figure 6:**
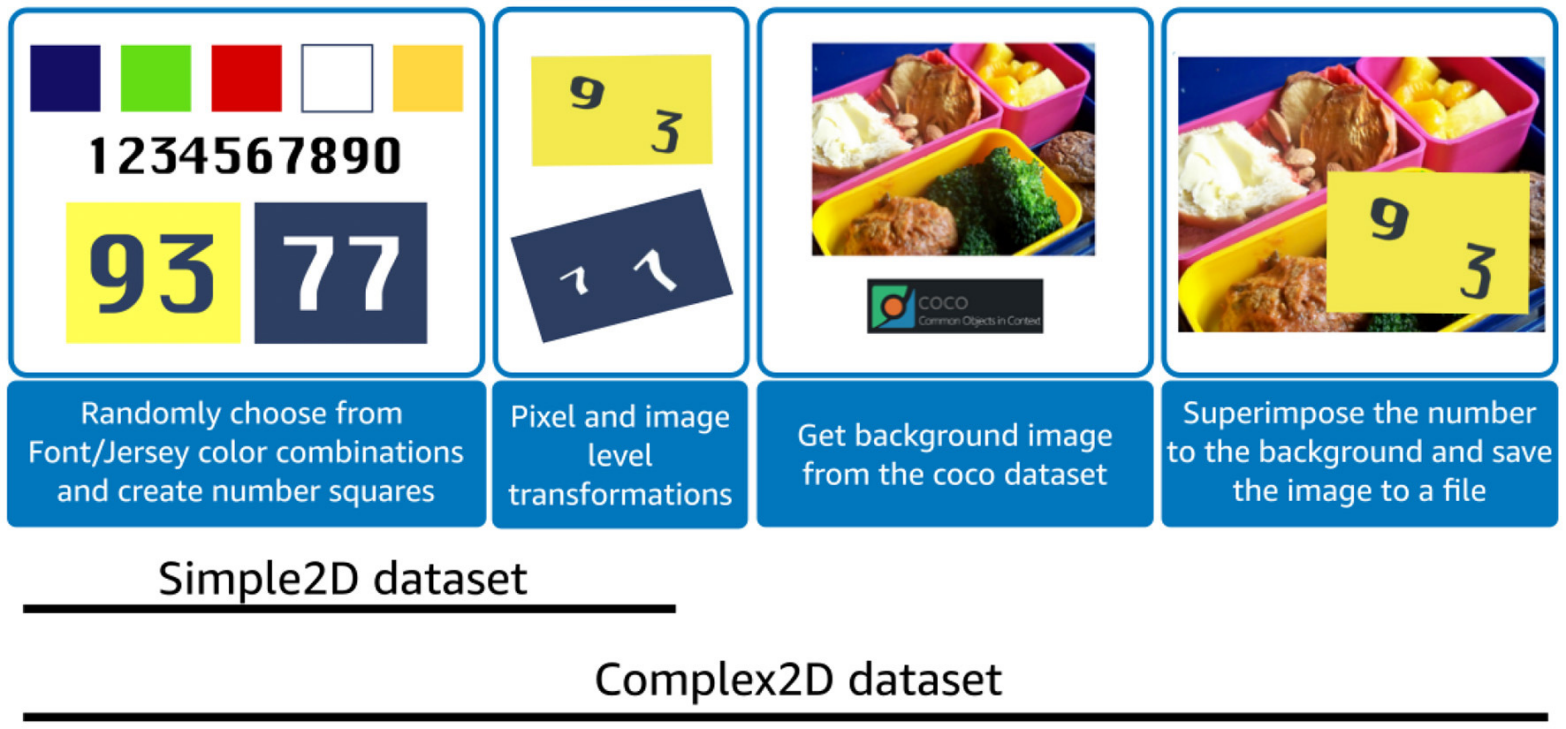
Synthetic data generation with Simple2D and Complex2D. Simple2D dataset was generated by creating numbers in football dataset jersey colors and fonts. Several augmentations (Table 1) were applied on these numbers to get Simple2D dataset. The numbers from this dataset were randomly sampled and randomly placed on COCO dataset images to form Complex2D dataset.

Since the real-world images had more complicated background, textures, and lighting conditions, we decided to synthetically generate another dataset (see Figure 6) to increase the robustness and generalization of our pre-trained model. The complex2D dataset was designed to increase background noise by superimposing numbers from Sample2D on random real-world images from the COCO dataset (Lin et al., 2014). We generated a total of 400,000 images (4,000 per class) with noisy backgrounds. Our algorithm is explained in more detail in Algorithm (1), Algorithm (2), and Algorithm (3).

**Algorithm 1:**
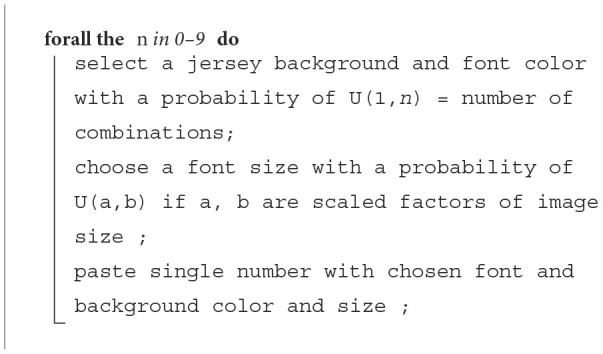
Number generation.

**Algorithm 2:**
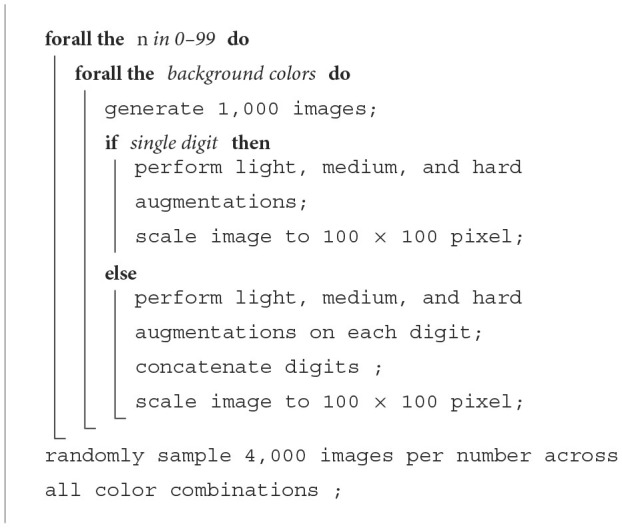
Simple2D.

**Algorithm 3:**
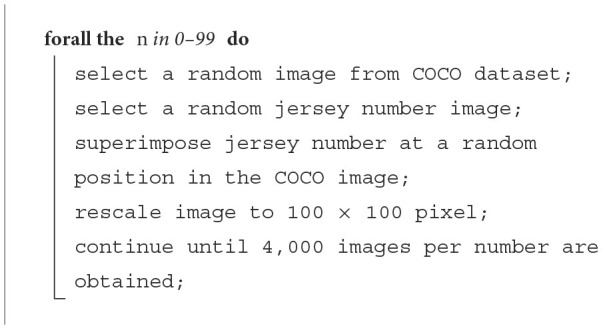
Complex2D.

#### 3.2.3. Jersey number detection

**Figure 7:**
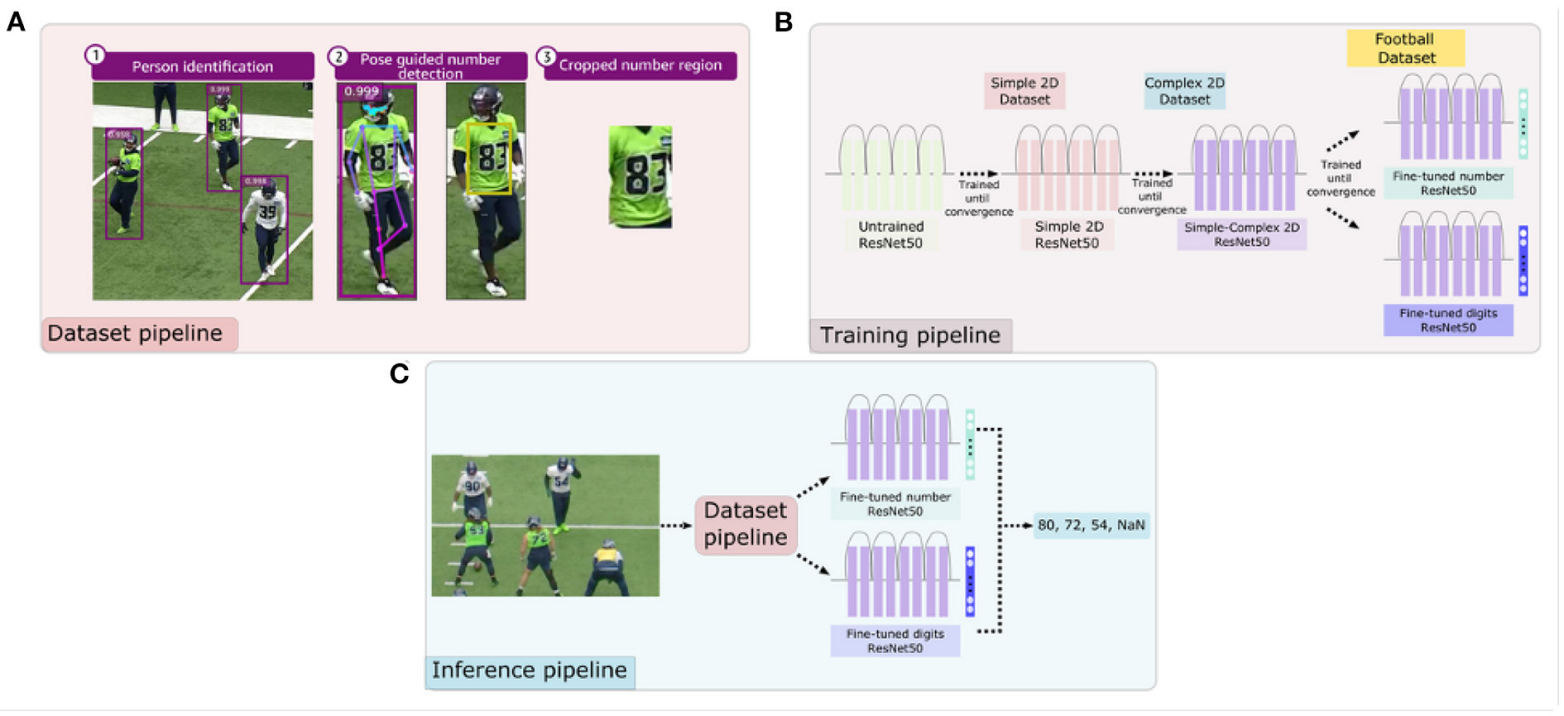
Overview of the approach for extracting data, training, and generating jersey number predictions. Panel **(A)** describes the high-level football dataset processing pipeline—identify person in video, pass each person image through the pose estimation model to identify the torso region and crop them. Panel **(B)** shows the sequential pre-training of multi-class/label models with synthetic number datasets—Simple2D and Complex2D as well as fine-tuning on football dataset. Panel **(C)** represents the inference pipeline that uses data pipeline from **(A)** to crop jersey numbers and perform prediction using multi-class/label models **(B)**.

After the number localization step above, two models were sequentially pre-trained with the synthetic datasets (Simple2D to Complex2D) and fine-tuned with the real-world football dataset (see Figure 7). The idea of training a model with increasingly difficult samples is called curriculum learning. This technique has empirically shown accuracy increase and faster convergence (Weinshall et al., 2018; Hacohen and Weinshall, 2019). One of the challenges of implementing curriculum learning is manually ranking difficulty in the training set (Weinshall et al., 2018). To address this challenge, the synthetic data was generated with increasing complexity and our training regime adopted this order.

Both models used a CNN architecture as backbone and a final layer predicting classes (jersey numbers). The first model was a multi-class image classifier to detect two-digit numbers with a total of 101 different classes (numbers from 0 to 99 plus an unrecognizable class). The second model was a multi-class multi-label classifier with 21 classes to detect single digits (10 digits for each side—right, left numbers, plus an unrecognizable class).

We define the *i*-th input feature *X*_*i*_ (cropped image of a player) with the label *y*_*i*_ (0–99 for actual numbers and 100 for unrecognizable). Our multi-class model was trained with the following loss function:


$$\begin{array}{l} L_{mc}=\underset{i}{\sum }L_{i}=-\underset{i}{\sum }y_{i}\log{ŷ}_{mc}\left(X_{i}\right) \end{array}$$

Where *y*_*i*_ is the true label and ŷ_*mc*_ is calculated as a softmax over scores computed by the multi-class model as follows:


$$\begin{array}{l} {ŷ}_{mc}\left(X_{i}\right)=\sigma \left(\vec{Z}\right)\\ \sigma {\left(\vec{Z}\right)}_{k}=\frac{e^{Z_{k}}}{\overset{100}{\underset{j=0}{\sum }}e^{Z_{j}}} \end{array}$$

Where $\vec{Z}$ is the output from the last layer of the multiclass model consisting of (*z*_0_, ..., *z*_1_00) given *X*_*i*_.

For the multi-label model, the loss function is defined as:


$$\begin{array}{l} L_{ml}=\underset{i}{\sum }L_{i}=-\underset{i}{\sum }y_{i}\log{ŷ}_{ml}\left(X_{i}\right) \end{array}$$

Where *y*_*i*_ is the true label and ŷ_*ml*_ is calculated as a sigmoid over scores computed by the multi-label model as follows:


$$\begin{array}{l} {ŷ}_{ml}\left(X_{i}\right)=\frac{1}{1+e^{\vec{Z}}} \end{array}$$

Where $\vec{Z}$ is the output from the last layer of the multilabel model given *X*_*i*_.

Both models were trained until convergence and the model from the epoch with the best performance was selected. We explored the combination of the two models to provide the final decision and we explain our results in section 4. Our hypothesis was that the multi-label model would augment performance of the multi-class model and address generalization issues with unseen or low data availability for certain numbers. For example, if 83, 74 were present in the training set but not 73, the right and left side of prediction nodes for 3 and 7 would have been activated in the train set for all numbers starting and ending with 7 or 3 and hence the multi-label model would have enough samples to predict 73.

We investigated training a custom object detection model to identify single-digit numbers. The image classification approach outperformed the object detection model primarily due to lack of labeled bounding boxes, image quality and small size of localized jersey numbers (approximately 20 × 25 pixel).

## 4. Experimental results

We trained the backbone CNN multi-class(number-detection) and multi-label(digit-detection) jersey number classifiers on the football dataset to establish baseline performance without the synthetic data. For the multi-class model, we took the number with the highest softmax score as the prediction. For the multi-label model, we applied a threshold of 0.5 to both right and left predicted classes to get the output. Eventually we computed the final prediction from the output of the two models.

The baseline model accuracy was 80% for both models. We experimented with various input image sizes and found optimal accuracy at 224 × 224 pixel for the multi-class and 100 × 100 pixel for the multi-label model. Our dataset presented a high imbalance across several numbers where 24% of the numbers have less than 100 samples and only 5% reach the 400-sample mark (see Figure 3). Hence, we duplicated data points for each number to have 400 images in the training set when needed. Our training pipeline dynamically applies image augmentation so that no image is seen twice by the models, even when the base image is the same. We also up sample our test-set images to maintain 20 images per number.

After establishing the baselines, we investigated the effects of pre-training with the generated synthetic data on our model performance. Pre-training on the Simple2D dataset and fine-tuning on the football dataset, resulted in a performance improvement of 2% over the baseline (82%), for both, multi-class and multi-label models. However, pre-training on the Complex2D dataset and fine-tuning on the football dataset, resulted in 3% improvement on the multi-class model and 8% on the multi-label model. By pre-training on both Simple2D and Complex2D, we achieved 8.8% and 6% improvement above the baseline in multi-class and multi-label models, respectively.

The best multi-label model (Complex2D + Football dataset) had positive accuracy improvements on 74 classes, no change in accuracy in 19 classes, and negative change in accuracy in 8 classes (drop by 10%). The best multi-class model (Simple2D + Complex2D + Football dataset) had positive accuracy improvements on 63 classes, no change in accuracy in 21 classes, and negative change in accuracy in 17 classes (drop by 7%). In order to validate the hypothesis (section 3.2.3) that multi-label model could have better performance on numbers with less images, we compare its results with the best multi-class model on numbers with less than 50 images in the training set. We notice an average increase in accuracy of 18.5% for multi-class model and 20% for multi-label model before and after training on synthetic data, for these numbers. Despite larger gains in accuracy shown by the multi-label model, the absolute accuracy scores for these numbers were better for the multi-class model, 81% compared to 78% for the multi-label model (Supplementary Figures).

**Figure 8:**
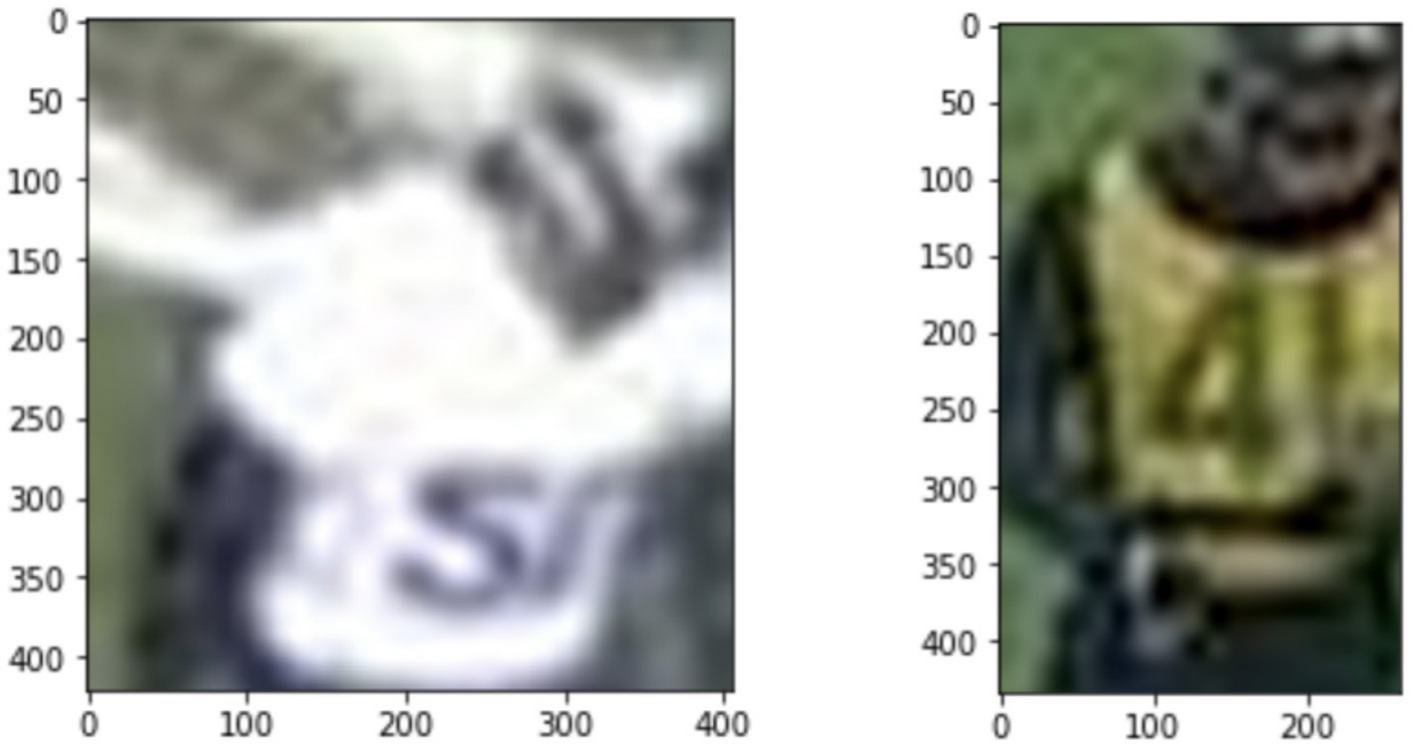
Images where multi-label predicted class 100. The multi-label model is not sure of the number class when the input image has very low resolution.

**Figure 9:**
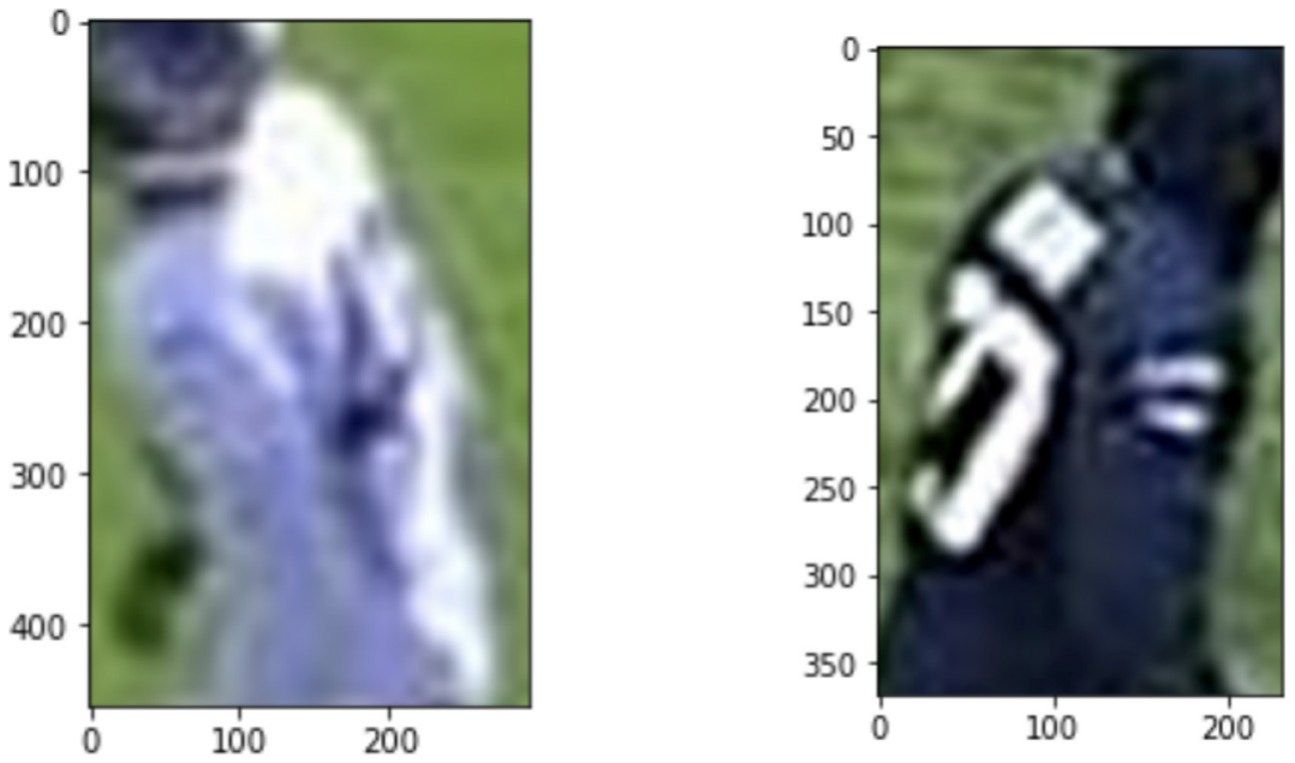
Image where multi-class predicted class 100. Confusion for the multi-class model arise when the numbers are rotated or occluded.

By analyzing the confusion matrix of the model predictions, we learnt that the best multi-label model produces false predictions in two major scenarios (see Figure 8): predicting one digit rather than both digits, and predicting class 100 for low-resolution and hard-to-recognize digits. In other words, the multi-label model is more likely to predict one digit number and non-number classes when challenged with new data. The multi-class model, however, has relatively spread-out false predictions (see Figure 9). Major areas of error for this model are: predicting one digit rather than both digits, and mistaking single digits for two digits or unrecognizable class.

Examining the performance of the two models independently we noticed that predictions agree in 84.4% of the test cases, suggesting that despite the different objectives (multi-class vs. multi-label) there is a robust learning of the number representations. Furthermore, we notice an additional improvement of 0.4% by two-model ensemble. Table 2 presents our results.

**Table 2:** A comparison of model performance under different conditions with confidence threshold of 0.5.

**Experiment**	**Multi-class**	**Multi-label**	**Ensemble**
**Without synthetic data**			
Football dataset	0.8064	0.8	
Best (Multi-class + Multi-label)			0.8028
**With synthetic data pre-training**			
Simple2D + Football dataset	0.8282	0.82	
Complex2D + Football dataset	0.8306	0.88	
Simple2D + Complex2D + Football dataset	0.8886	0.86	
Best (Multi-class + Multi-label)			0.8931

### 4.1. Comparisons with other number detection approaches

Our solution presents a method to detect jersey numbers in a low-data regime. We used ResNet50 as our backbone CNN model and proposed synthetic data generation methods to improve the results. We used ResNet50 because of the wide adoption in the ML community, efficient use of resources, and availability of the pre-trained model. We were able to achieve comparable results to the state of the art models with orders of magnitude more trainable parameters.

Table 3 demonstrates the comparison of published methods vs. our proposed approach.

**Table 3:** Summary of published methods in comparison to the proposed method.

**Dataset**	**Annotated data size**	**Annotations**	**Algorithm**	***n*_*params*_ (M)**	**Accuracy (%)**
Soccer matches premier league	12,746	Number class and region of interest sampling	CNN with spatial transformation block by Li et al. (2018)	–	86.7%
Soccer matches	3,567	Number bounding box, person bounding box, torso keypoints	Pose-guided object detection by Liu and Bhanu (2019)	41.5 M	90.44%
National Hockey team	38,456	Number class	Multi-task classification by Vats et al. (2021)	21.5 M	89.6%
American football	3,000	Number class	Our proposed three-step method	4.1 M	89.31%

*n*_*params*_ is the total number of trainable parameters.

## 5. Limitations

The work presented in this paper shows that the number identification task can be simplified by leveraging synthetic datasets. We were able to achieve a similar performance compared to previous works (Ye et al., 2005; Šari et al., 2008; Gerke et al., 2015) requiring no change in the data collection pipeline. Despite these findings, we recognize this approach has some limitations which we describe in this section.

We were able to achieve 89% accuracy for our test dataset despite the challenging nature of jersey number identification in a low-data regime. This performance is on par with some of the most recent works (Vats et al., 2021). However, the lack of benchmark datasets for this task and unavailability of tools, is an crucial barrier for comparing performance across all methods. The only solution is to label large amounts of high-quality data and retrain the available solutions in-house. This requires a lot of computational resources and manual effort to work, which is not an option for all institutions.

In our jersey detection models, we used ResNet50 as our base model primarily because of the wide adoption in the ML community, efficient use of resources and availability of the pre-trained model. Bigger and more sophisticated models might provide better accuracy and recall but they come with extra environmental and financial costs. We recognize that more investigation is needed here to determine the optimal solution.

In our solution we chose a three-model pipeline approach vs. a one-pass prediction model. Our approach comes with a few limitations including cascading inaccuracies from one model to the next and increase in latency. However, our choice was justified by ease of implementation, maintenance and portability to other domains. Even with this cascading effect, our solution proves to have a good performance in our highly imbalanced, limited dataset.

## 6. Future work

Our approach to increase performance can be broadly classified into two categories: improving data quality and quantity or experimenting with different models.

### 6.1. Data quality and quantity

We observed no improvement in model accuracy by increasing the number of duplicated samples or the number of image augmentations. The confidence of the predictions directly correlated with the quality and resolution of the jersey number crop (input image). In our future works, we plan to experiment with various image quality enhancement methods in classical CV and deep learning domains to observe if it improves performance. Another path that can be considered is to refine our synthetic data generation pipeline to produce images that are closer to the real-world dataset.

### 6.2. Different model strategies

Our current method has minimal labeling effort. However, by collecting more images of reasonable quality and quantity we plan to test object detection-based models. One way to improve frame level accuracy would be to track detected jersey numbers across both side-line and end-zone views so that in situations where numbers are partially visible or player pose is complex, we would be able to obtain predictions with continuity. Tracking players in team sports like football is still a major challenge in the sports CV domain and we will evaluate its utility in our future works.

## 7. Conclusion

This paper presented a new solution for low-data regime jersey detection with two-stage novel synthetic data generation techniques, pose estimation for jersey number localization and CNN ensemble learning to detect jersey numbers. Data augmentations during training and the use of large synthetic dataset provided enough variations for the model to generalize well and learn numbers. Our solution is easy to implement, requires minimal labeling, curation, supervision, and can be customized for various sports jersey fonts, colors, and backgrounds. Our framework improves the accuracy of the number detection task by 9% and can be easily extended to similar tasks across various Sports communities as well as industries with similar use cases. Furthermore, our solution did not require the modification of the data capturing or processing pipeline that is already in place, making it convenient and flexible.

Additionally, it introduces a novel data synthesis technique that can boost custom solution performance in a wide array of sports. We hope this solution enables the Sport Analytics community to rapidly automate video understanding solutions.

## Data availability statement

The datasets presented in this article can be made available at request. Data access requests should be directed to erpelaez@amazon.com.

## Author contributions

DB: conceptualization, methodology, software, formal analysis, investigation, data curation, writing—original draft, writing—review and editing, and supervision. EP: conceptualization, methodology, software, investigation, data curation, writing—original draft, writing—review and edit, visualization, and supervision. SG: investigation, project administration, writing—original draft, and writing—review and editing. All authors contributed to the article and approved the submitted version.

## Funding

This work was developed by Amazon Web Services employees under AWS infrastructure.

## Conflict of interest

Authors Db, SG, and EPC were employed by company Amazon Web Services. The authors declare that this study received funding from Amazon Web Services. The funder had the following involvement with the study: approval of the manuscript.

## Publisher's note

All claims expressed in this article are solely those of the authors and do not necessarily represent those of their affiliated organizations, or those of the publisher, the editors and the reviewers. Any product that may be evaluated in this article, or claim that may be made by its manufacturer, is not guaranteed or endorsed by the publisher.
