# Supplementary figures and images for: Jersey number detection using synthetic data in a low-data regime
Source: Front Artif Intell. 2022 Oct 6;5:988113. doi: 10.3389/frai.2022.988113 (PMC9583843; doi:10.3389/frai.2022.988113)

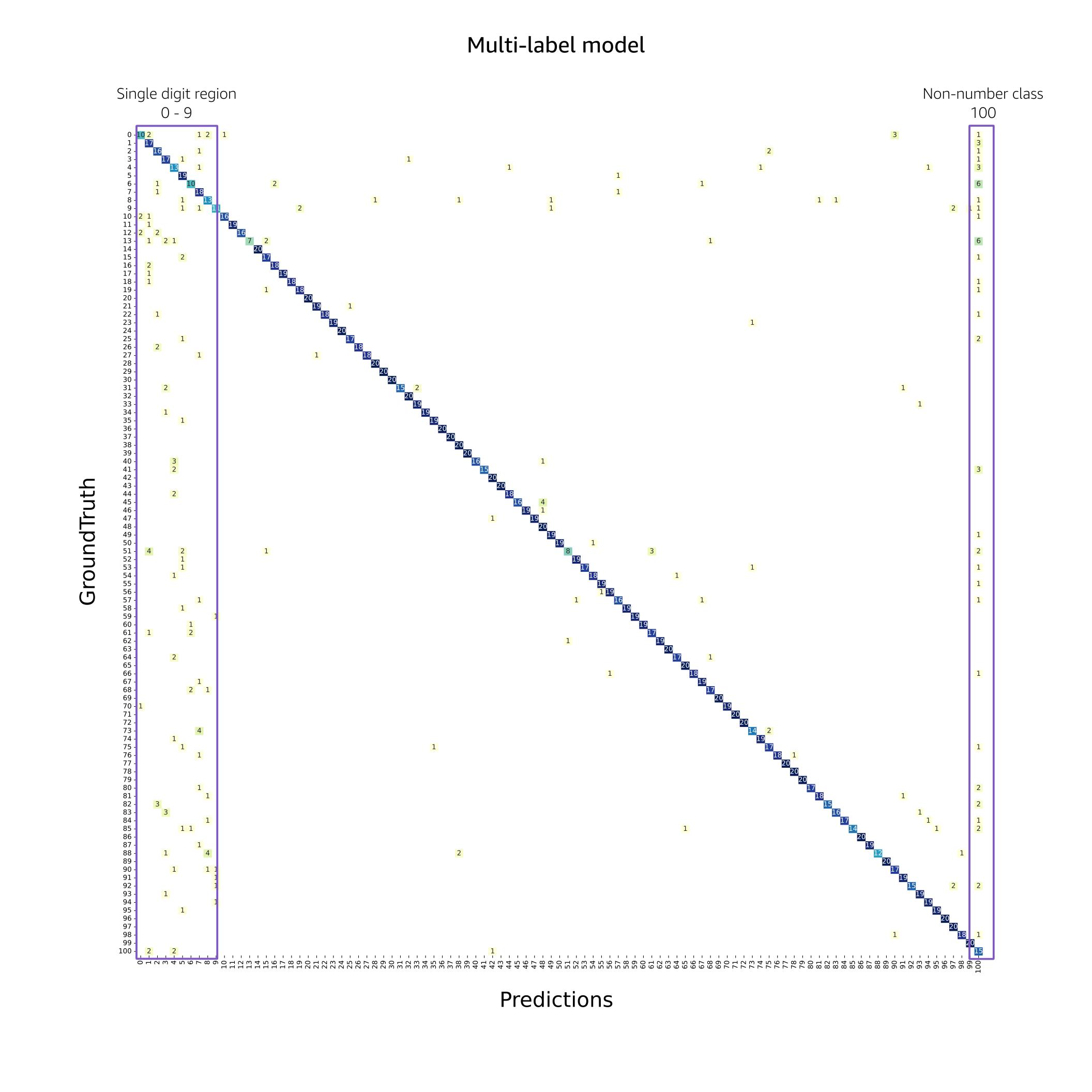


Figure 1: Multi-label confusion matrix


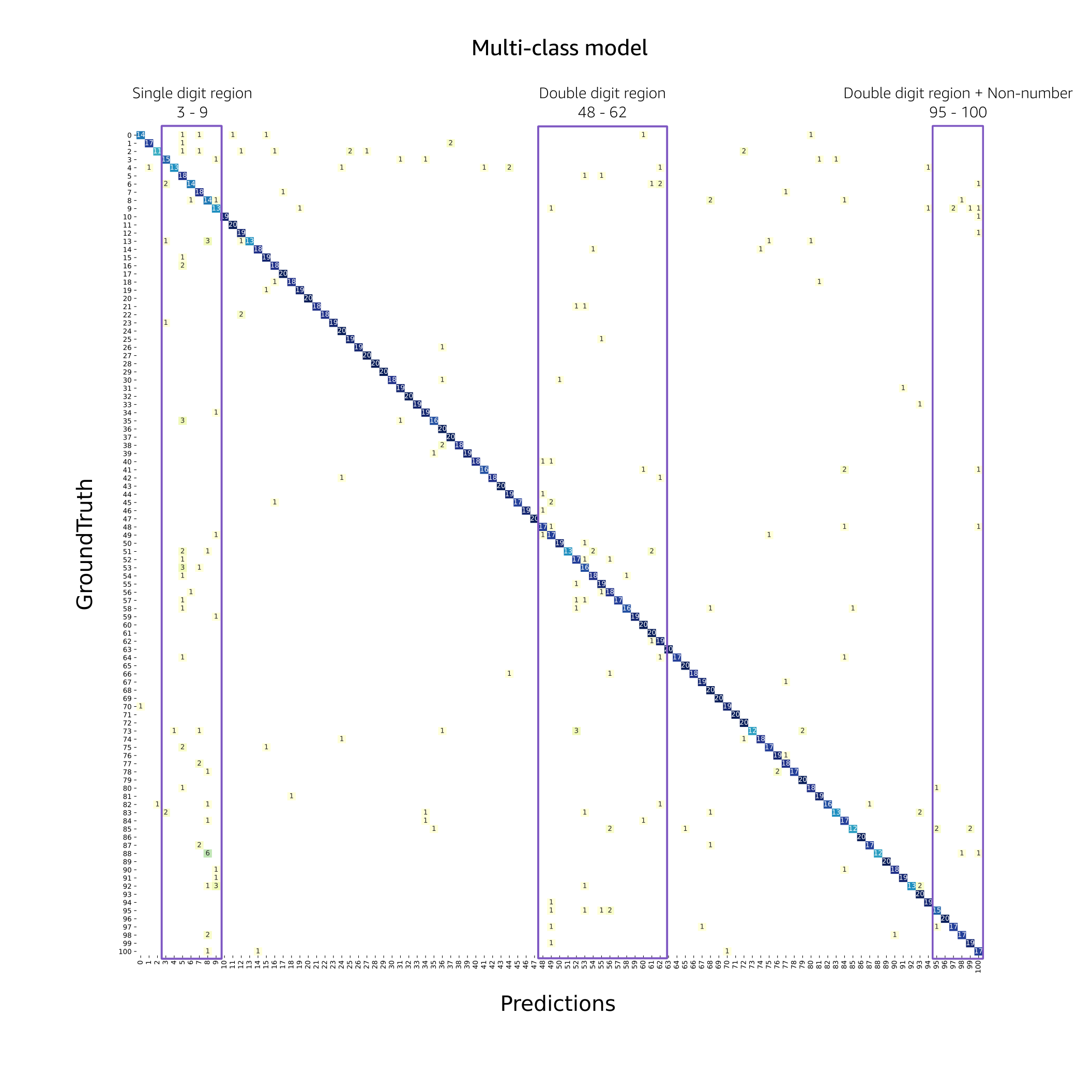


Figure 2: Multi-class confusion matrix.

Supplement: Supplementary file 1 [file Data_Sheet_1.docx]
